# Comparison of Joint Loading in Badminton Lunging between Professional and Amateur Badminton Players

**DOI:** 10.1155/2017/5397656

**Published:** 2017-06-13

**Authors:** Lin Fu, Feng Ren, Julien S. Baker

**Affiliations:** ^1^Faculty of Sports Science, Ningbo University, Ningbo 315211, China; ^2^School of Science and Sport, University of the West of Scotland, Hamilton ML3 OJB, UK

## Abstract

The knee and ankle are the two most injured joints associated with the sport of badminton. This study evaluates biomechanical factors between professional and amateur badminton players using an injury mechanism model. The aim of this study was to investigate the kinematic motion and kinetic loading differences of the right knee and ankle while performing a maximal right lunge. Amateur players exhibited greater ankle range of motion (*p* < 0.05, *r* = 0.89) and inversion joint moment (*p* < 0.05, *r* = 0.54) in the frontal plane as well as greater internal joint rotation moment (*p* < 0.05, *r* = 0.28) in the horizontal plane. In contrast, professional badminton players presented a greater knee joint moment in the sagittal (*p* < 0.05, *r* = 0.59) and frontal (*p* < 0.05, *r* = 0.37) planes, which may be associated with increased knee ligamentous injury risk. To avoid injury, the players need to forcefully extend the knee with internal rotation, strengthen the muscles around the ankle ligament, and maximise joint coordination during training. The injuries recorded and the forces responsible for the injuries seem to have developed during training activity. Training programmes and injury prevention strategies for badminton players should account for these findings to reduce potential injury to the ankle and knee.

## 1. Introduction

Badminton, a popular racquet sport characterised by a hand-held racquet used to propel a missile (shuttlecock) between players [1], has been commercialised and popularised for athletic competitions and recreational physical activities [2]. Over 200 million players of different ages, genders, and skill levels [3] participate. The lunge is a critical movement in badminton, which enables players to quickly move into the best position for the next shot, followed by a return to the start position or move off into another direction for the next movement [4, 5]. Previous studies about competitive badminton reported that the lunge represented over 15% of all the movements in a single match [5]. An important determinant of lunge performance is the ability to move quickly with power (time to peak force) excluding factors such as body mass, flexibility, and leg length [4, 6].

The lunging step is not exclusive to badminton and also plays an important role in other sports such as tennis, squash, and fencing [1, 4, 5]. A fast and accurate lunge via fencing analysis has been shown to constitute a pivotal position in a successful fencing attack [7] and promotes tactical uncertainties for the opposing fencer [8].

The lunge is also used as a functional movement assessor of muscular strength in tennis [9]. Furthermore, the forward lunge has been used as a functional test for anterior cruciate ligament (ACL) deficiency and knee stability [10, 11]. Research shows that badminton players require high strength ability, in addition to the special technical requirements to play the game. These requirements are greater than just the local strength and ability to perform in a competitive environment. The survey found that amateur players generally placed more emphasis on technical training, but did include some local strength training that was associated with technique. If the ability of the individual is low and the quality of training is poor, the exercise duration becomes too long or repetitive. This in turn results in increases in practice time, practicing specific skill requirements. Therefore, the extra training time, and the forces generated during the training period make it difficult for the lower limbs to withstand the load, which may cause injury.

Lees [1] reported that kinematic metrics have primarily been used to assess the mechanisms underlying performance for racquet sports, and there has been less importance placed on kinetics of racquet sports [1, 5]. In particular, less emphasis has been placed on loading of the lower extremity joints, which are closed dynamic chains for weight bearing [12]. It has been reported that stress accumulates in both the Achilles tendon and anterior knee tendons in professional badminton players after competition, particularly the dominant lunge leg [13]. It has been suggested that loading was closely related with the risk of injury, although racket sports were believed to have lower injury rates [1, 5]. Furthermore, most injuries affect competition and training regimes [14, 15]. Clinical analysis of injuries in racquet sports were mainly focused on the lower limb (over 58%), especially the knee and ankle [13, 16–19].

Bahr and Krosshaug [16] proposed a comprehensive injury causation model based on the epidemiological model of Meeuwisse [20] from a biomechanical perspective [21] and sport-related characteristics, which included intrinsic risk factors, extrinsic risk factors, and an inciting event. Previous studies related to badminton injuries showed that almost all the injuries occurred during training or competition, and joints (ankle and knee) of the lower limb were the dominant injury site [13–15, 18, 19, 22]. External leading factors, like partner collision, being struck by a racket or shuttlecock, and issues from badminton court or shoes, accounted for only a small percentage of total badminton injuries [3, 19, 23]. The foremost factors of badminton-related injuries are intrinsic, taking the actions of moving into and returning the shuttlecock or in the racquet stroke as examples [4, 22, 24, 25].

Kinetic analysis of racquet sports, such as badminton, may provide biomechanical mechanisms to explain the high rate of lower limb injuries reported clinically, especially between different levels of players. However, previous studies investigating badminton movement mainly focused on different movements or lunge movement directions without comparing movement characters between professional and amateur badminton participants [24–28]. In this study, we hypothesised that amateur and professional badminton players exhibit differences in the forehand right-forward lunge movement and these differences in kinetics and joint moments make them prone to different injury mechanisms. This study aimed to investigate the kinematic motion and kinetic loading of the dominant leg joints, compared with the nondominant side. A second aim was to investigate if the dominant leg was stronger, with a greater range of movement (ROM). The increase in strength and ROM which facilitates movement patterns and technique may increase the risk of injury for both professional and amateur players.

## 2. Materials and Methods

### 2.1. Ethics Statement

The test was approved by the Ethics Committee of the Faculty of Sports Science in Ningbo University. Written consent was obtained from all the participants and they were informed of the procedures and requirements of the lunge test.

### 2.2. Participants

A total of sixteen male participants took part in the study, including eight professional badminton players (ages: 23.4 ± 1.3 yrs; height: 172.7 ± 3.8 cm; mass: 66.3 ± 3.9 kg; badminton playing years: 9.7 ± 1.2 yrs), who were members of the province club and participated at professional national level, and eight amateur badminton players (ages: 22.5 ± 1.4 yrs; height: 173.2 ± 1.8 cm; mass: 67.5 ± 2.3 kg; badminton playing years: 3.2 ± 1.1 yrs), who competed for their college or university in intercollegiate play. There were no significant differences in demographic data between the two groups. The right hand and leg was dominant for all participants. The professional badminton players were recruited from the local badminton clubs and the amateur badminton players were voluntary badminton-major students from the Faculty of Sports Science in Ningbo University. All participants were free from any injuries to the upper and lower limbs in the six-month period preceding the study. The subjects also refrained from any high-intensity training or competition two days prior to testing.

### 2.3. Procedures

**Figure 1:**
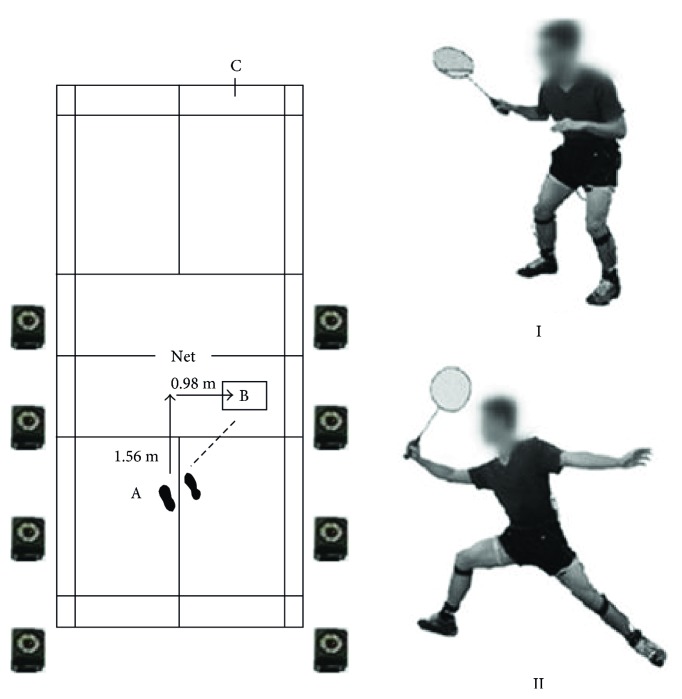
The illustration of simulated badminton court (a) and preparatory (I) and lifting shuttlecock (II) positions (b).

The test was conducted in a lab-based badminton court with an eight-camera Vicon® motion capture system (Vicon MX, Vicon Motion System Ltd., Oxford, UK) and Kistler® force platform (Kistler Type, 9281B, Kistler Instrument AG, Winterthur, Switzerland) with size of 600∗900 mm.  Figure 1 shows positions A, B, and C, respectively, representing the starting position, force platform, and landing area of the shuttlecock. The simulated court surface was covered with dedicated flooring of a professional standard. Prior to the test, participants were required to perform a ten-minute warm-up for court familiarisation. The shuttlecocks were thrown to position B from the other half court, consistently delivered at the same height. Participants were required to perform maximal right forward lunge from position A (start/finish position) and to underhand stroke the shuttlecock to the backcourt (position C; see Figure 1). After striking the shuttlecock, participants were required to return to position A. This was in accordance with the movements reported by Huang et al. [24]. During the test, all participants wore the same brand and series of badminton shoes, avoiding any influence from badminton footwear with different material properties [23].

### 2.4. Data Collection

Kinematic data were collected using an eight-camera Vicon motion capture system at a frequency of 200 Hz, based on previous experimental Plug-in-Gait protocols [24, 25, 28, 29], which ensured the validity and reliability of the tests. The marker set included sixteen markers (diameter of 14 mm), bilaterally located to the anterior-superior iliac spine, posterior-superior iliac spine (PSI), lateral thigh (THI), lateral knee (KNE), lateral tibia (TIB), lateral ankle (ANK), heel (HEE), and toe (TOE) [24, 25]. Measurements were taken for the calculation of joint centres: height, weight, leg length, and width of the knee and ankle. Before the dynamic measurement, participants were required to stand still for the participant-specific static model calibration using Vicon software Nexus 1.8.5, so as to define lower limb joint motion axes and planes. The stance was defined as right foot landing onto the force platform until right foot-off the platform using the vertical ground reaction force (vGRF) from a Kistler force platform at a frequency of 1000 Hz, which was similar with the vGRF pattern of the kick lunge task reported by Kuntze et al. [5].

### 2.5. Data Analysis

**Figure 2:**
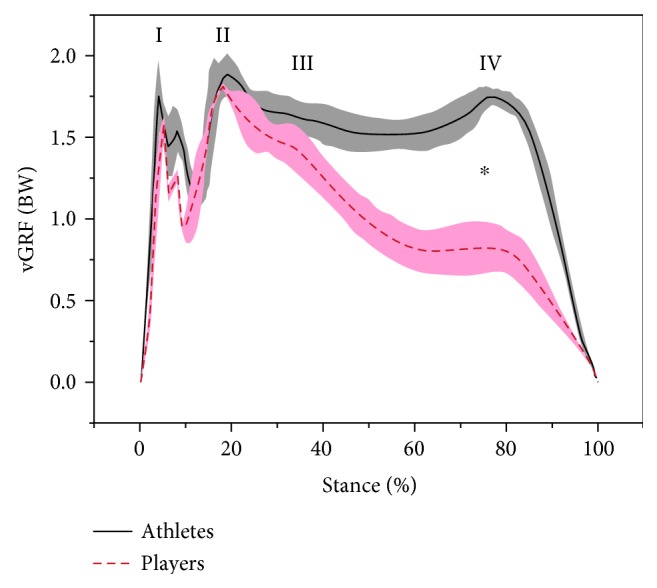
The illustration of mean vertical ground reaction force (vGRF) (with standard deviation) pattern of badminton players in the stance of lunge (I, II, III, and IV, resp., indicate the initial impact peak, secondary impact peak, weight acceptance, and drive-off phases; ∗ indicates significant difference at *p* < 0.05).

Ground reaction forces and kinematic data were collected simultaneously during the experiment. For each participant, the kinematic and kinetic data of six successful trials were averaged for analysis. The range of motion (ROM) of the knee and ankle in three dimensions (sagittal, frontal, and horizontal planes) were measured during stance, and the knee and ankle joint moments were calculated using a three-dimensional inverse dynamics approach. For the stance period, the contact and lift-off were determined from the vGRF magnitude at 20 N. The stance was divided into four phases, including initial impact peak (I, 5% of stance), secondary impact peak (II, 20% of stance), weight acceptance (III, 40%–70% of stance), and drive-off (IV, 80% of stance) (Figure 2). Drive-off phase can get enough time to keep the body balanced to reduce the injury. The ROM of the ankle and knee were calculated from the maximum and minimum joint angles in the three-dimensional motion planes. All joint moment data were normalised using participants' body weight in Newtons (N).

### 2.6. Statistical Analysis

SPSS 17.0 software (SPSS for Windows, Chicago, IL, USA) was utilised for statistical analysis. The collected ankle and knee joint ROM data and calculated joint moments were tested using independent-sampled *t*-tests between badminton athletes and players. The power analysis was calculated using NCSS-PASS 11.0.7 software. A two-sample *t*-test was used to calculate the appropriate number of subjects to ensure that data analysis satisfied statistical power. Joints' ROM and moment values were indicated with mean, standard deviation, mean difference (MD), and 95% confidence interval (CI). The significance level was set at *p* = 0.05.

## 3. Results

The mean (standard deviation shown with shaded bars) values (normalised to body weight) of the vertical ground reaction forces (vGRF) are illustrated in Figure 2. There were statistically significant differences in the drive-off (IV) phase between professional and amateur badminton players (*p* < 0.05).

The ankle and knee range of motion (ROM) during the stance are shown in Table 1. There was a statistically significant difference in the frontal ((*p* = 0.002 < 0.05), MD = −3.12, 95% CI = [−4.54, −1.71]) and horizontal ((*p* = 0.048 < 0.05), MD = −5.67, 95% CI = [−11.27, −0.06]) planes of the ankle. Amateur badminton players showed greater ROM in the frontal plane (inversion/eversion movement), but smaller ROM in the horizontal plane (external/internal rotation movement). Professional badminton players also showed a greater external/internal rotation movement of the knee in the horizontal plane ((*p* = 0.000 < 0.05), MD = −13.51, 95% CI = [−17.23, −9.79]) than amateur badminton players.

In addition to the statistically significant results, professional badminton players showed trends for larger ROM in the sagittal plane (dorsiflexion/plantar flexion and flexion/extension) of the ankle and knee and frontal plane (adduction/abduction) of the knee.

**Figure 3:**
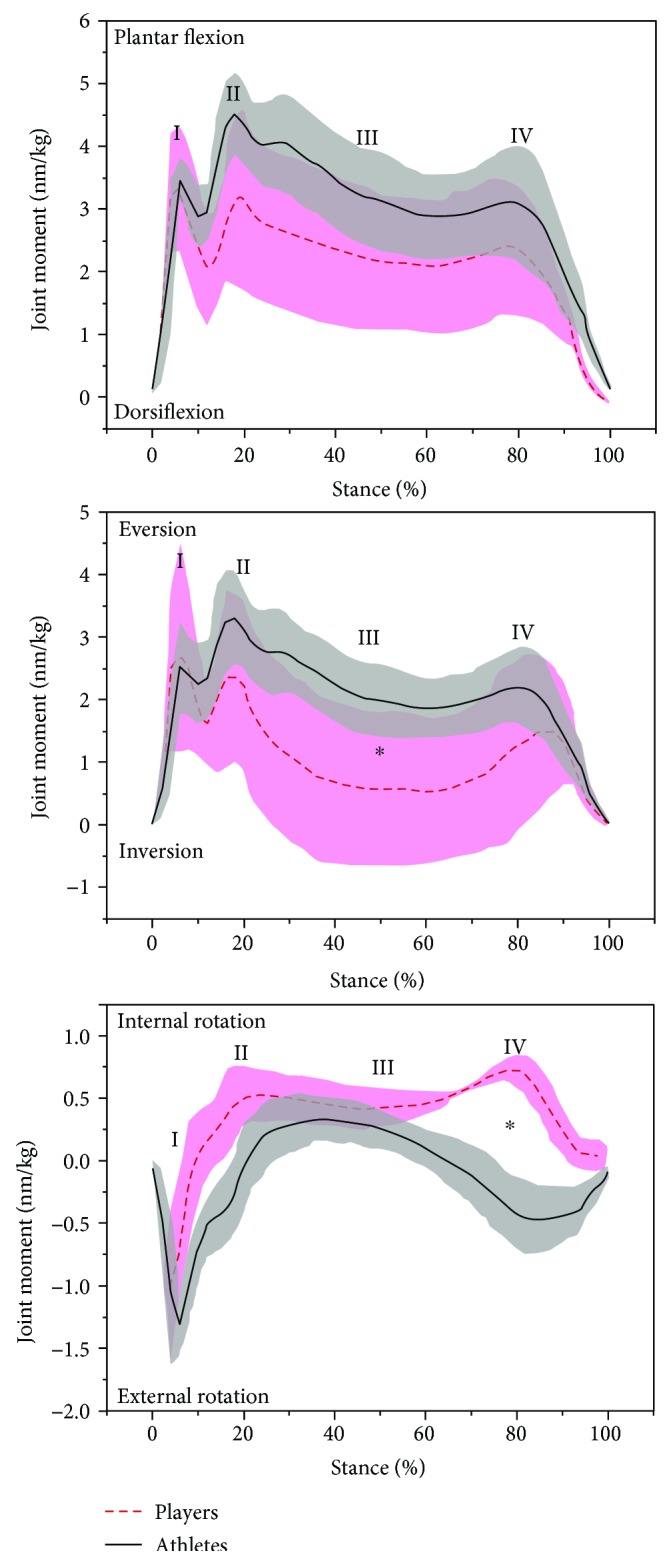
The mean (standard deviation—shadow with bars) value of ankle joint moment in the sagittal (plantar flexion/dorsiflexion), frontal (eversion/inversion), and horizontal (internal/external rotation) planes between professional and amateur badminton players (∗ indicates significance level *p* < 0.05).

The ankle joint moments in the three-dimensional planes are illustrated in Figure 3. Amateur badminton players showed smaller eversion moment ((*p* < 0.05), MD = −0.91, 95% CI = [−1.19, −0.63]) (or bigger inversion moment) in the weight acceptance phase of lunge stance and larger internal rotation moment ((*p* < 0.05), MD = 0.47, 95% CI = [0.33, 0.62]) in the drive off phase of lunge stance.

**Figure 4:**
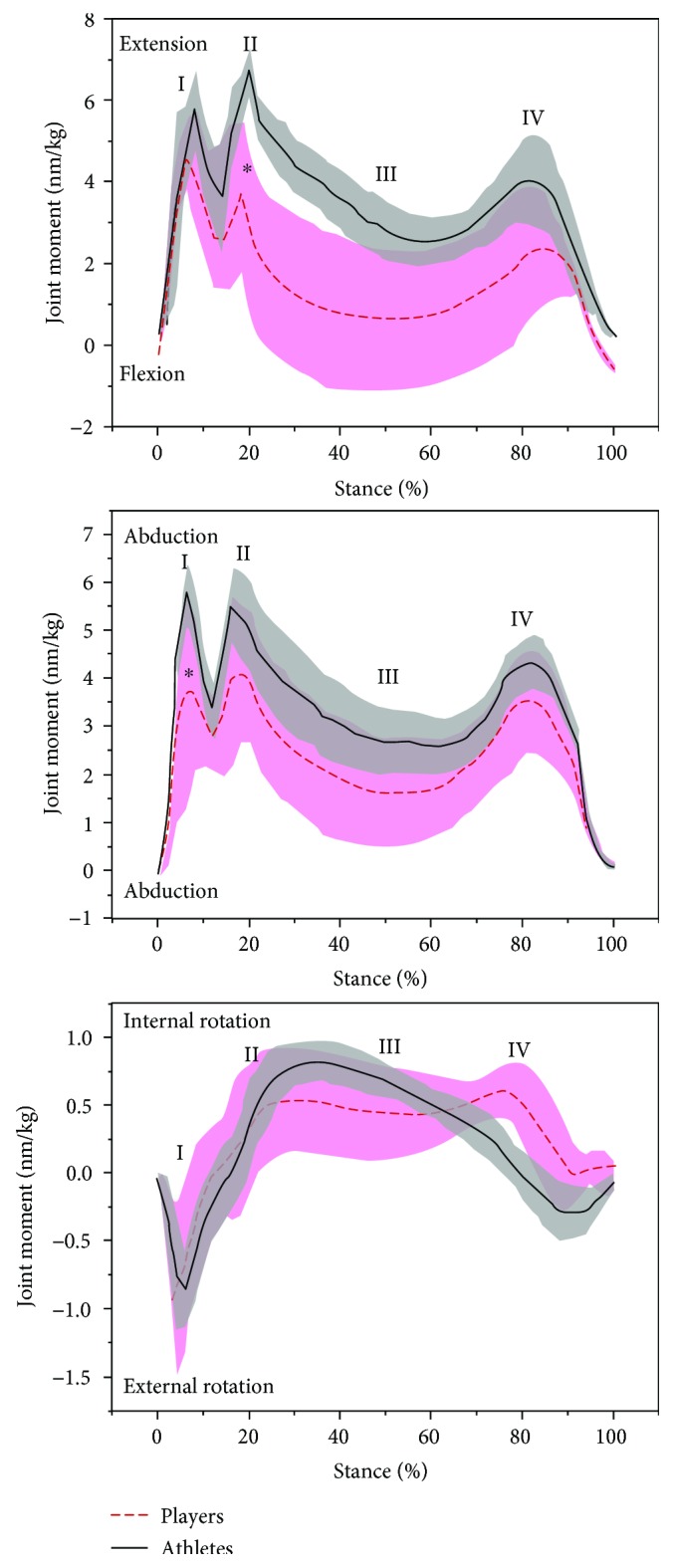
The mean (standard deviation—shadow with bars) value of knee joint moment in the sagittal (extension/flexion), frontal (abduction/adduction), and horizontal (internal rotation) planes between professional and amateur badminton players (∗ indicates significance level *p* < 0.05).

The knee moments are shown in Figure 4. Professional badminton players showed significantly larger extension moment ((*p* < 0.05), MD = −1.87, 95% CI = [−2.36, −1.38]) in the secondary impact peak phase in the sagittal plane and greater abduction moment ((*p* < 0.05), MD = −0.9, 95% CI = [−1.37, −0.44]) in the initial impact peak in the frontal plane (Figure 4).

## 4. Discussion

Increased sport participation, while beneficial for health, is associated with increased injury risk for both professional and amateur players [30]. Hence, knowledge of injury mechanisms is central to injury prediction, prevention, and treatment [20]. Specifically, the multifactorial approach including internal and external risk factors and inciting event has been reported as effective [16, 20, 31], especially for knee and ankle injuries in many sports, including badminton [13–15, 18, 19, 22].

Previous studies have evaluated the biomechanical characteristics of the badminton lunging step [5, 24, 25] without considering the badminton performance (skill level) of the player in their biomechanical analysis. In this study, we have shown that this is important as professional badminton players and amateur badminton players showed different joint ROM and joint torques while performing a forehand right-forward lunge.

Considering the vGRF patterns of professional and amateur badminton players, the stance could be divided into four phases, including initial impact peak (I, 5% of stance), secondary impact peak (II, 20% of stance), weight acceptance (III, 40%–70% of stance), and drive-off (IV, 80% of stance) (Figure 2). This was consistent with the previous published kick lunge task by Kuntze et al. [5] and other lunging step studies [32]. Moreover, the difference observed during the drive-off phase, between professional and amateur badminton players, may be attributed to badminton athletes having stronger knee extensors, including the vastus lateralis, vastus medialis, and rectus femoris muscles [4, 25]. Badminton athletes also exhibited generally greater extension moment; however, these results did not reach a level of significance (Figure 4).

Amateur badminton players showed significantly larger ROM in the frontal plane than professional badminton players, which may be linked with poor muscle (peroneal muscles) strength around ankle instability [4, 33, 34]. Integrated with ankle eversion/inversion moment (Figure 3), the smaller eversion moment (or larger inversion moment) of amateur badminton players may present a latent lateral ankle sprain injury risk, particularly phase III. During body weight transfer of the whole foot of the forward lunge leg, the greater ankle ROM and ankle's smaller eversion moment in the frontal plane may contribute to ankle inversion sprain [34, 35]. Furthermore, the external rotation ankle moment in the horizontal plane at phase IV for professional badminton players is in contrast to the internal rotation moment exhibited by amateur badminton players and reveals different ankle stability mechanisms.

The total lunge movement requires coordination of the lower extremity, trunk, and upper extremity movements. This centres around the dynamic stability of the knee joint as a crucial factor for performance and injury prevention [5, 24, 25]. The greater ROM of professional badminton players could be attributed to a better and injury-preventive foot landing technique during the stance phase [3, 6]. Furthermore, lunge motions require a large knee movement to transfer the whole body centre of mass (COM) to maintain balance [25]. As shown in Figure 4, professional badminton players showed significantly higher knee joint moments extension in phase II and greater abduction moments in phase I. The higher knee joint moments of professional badminton players may increase the risk of knee injuries demonstrated by previous studies [13, 36]. Both phase I and phase II, body weight was transferred to the knee. Sport-related injuries result from hyperflexion of the knee, this mechanism is the most common cause of isolated PCL injuries. These injuries often have greater instability because of the greater joint moments in sagittal and frontal planes, and they could be injury risk factors for knee internal cruciate ligaments (anterior cruciate ligament (ACL) and posterior cruciate ligament (PCL)), the collateral ligaments, and the meniscus [12, 36–38]. This finding may play a role in injury identification as injured professional badminton players would show smaller ROM at the knee because of the conservative movement pattern to minimise pain and injury recurrence [24, 25].

The kinematic and kinetic findings in this study show consistency with previous studies of badminton lunge biomechanics. Such as the finding demonstrating knee pain was related to knee motion during badminton forehand lunge [24].

In addition, a study comparing a control group and knee-injured group while performing a right forward lunge outlined similar knee ROM to the findings presented here [25]. For the kinetic results, findings about knee joint moments were higher here than the results of Lam et al. [27] and Kuntze et al. [5]. This could be explained by the fact that participants in this study were required to perform a maximal lunge, which was linked with greater forces. Previous studies have focused on different movements, like single lunge or repeated lunge movements [27]; kick, step-in, and hop lunge movements [5]; and lunge movement in directions (forehand or backhand forward and backward) [24, 25]. These studies have found similar findings to those reported in this study and therefore have complimented the validity and reliability of the results presented here.

A widely accepted objective in sport participation is to improve performance and minimise injury risk to lengthen sport careers for both professional and amateur badminton players [15, 17, 30, 34]. For amateur players, a comprehensive exercise scheme encouraging correct technique, particularly the foot landing position in stance due to the higher injury during plant-and-cut action [36], is suggested. For professional badminton players, the higher loading (joint moment) to the knee should be considered by players and coaches with regard to ligament overuse injuries. Loading alleviation-related protection braces and athlete-specific badminton footwear for the minimisation of ligament injury may be useful [23, 32].

A limitation of this study was the small sample size, which should be enlarged in future studies. It should also be acknowledged that when interpreting the findings from this study the lower extremity muscle activity of professional and amateur badminton players was not collected during the lunge test. Muscle activation and strength will also play a key role in explaining the differences reported between professional and amateur badminton players. Future studies should evaluate different movement characteristics of different skill level participants integrating joint loading and muscle activity together.

## 5. Conclusion

Findings from this study highlighted that different internal injury risks exist between professional and amateur badminton players. Amateur badminton players exhibited greater ankle range of motion and inversion joint moment in the frontal plane as well as greater internal joint rotation moment in the horizontal plane. These kinematic and kinetic differences when compared with professional badminton players may represent a poor grasp of the right lunge landing technique and increase the likelihood of ankle inversion sprain injury. In contrast, professional badminton players presented a greater knee joint moment in the sagittal and frontal planes, which may be associated with increased knee ligamentous injury risk. Training schemes and injury prevention strategies for amateur badminton players should account for these findings to reduce potential injury to the ankle and knee.

## Figures and Tables

**Table 1:** ROM (range of motion) of ankle and knee in the stance phase of lunge (degrees).

		Sagittal	Frontal	Horizontal
Ankle	Professional players	26.05 (5.55)	3.69 (0.15)	24.07 (4.40)
	Amateur players	18.95 (3.25)	6.81 (1.13)	18.40 (0.78)
	95% CI	[−15.57, 1.38]	[−4.54, −1.71]	[−11.27, −0.6]
	*p*	0.084	0.002^∗^	0.048^∗^
	Power	0.820	1.000	0.875

Knee	Professional players	66.35 (4.41)	22.87 (5.15)	35.44 (1.70)
	Amateur players	62.22 (5.97)	18.51 (3.79)	21.93 (2.01)
	95% CI	[−14.74, 6.47]	[−12.97, 4.25]	[−17.23, −9.79]
	*p*	0.362	0.25	0.000^∗^
	Power	0.313	0.437	1.000

Notes: sagittal, frontal, and horizontal planes represent the flexion/extension (knee) and dorsiflexion/plantar flexion (ankle), varus/valgus (knee) and inversion/eversion (ankle), and external/internal rotation (knee and ankle). 95% CI: 95% confidence interval; ∗ indicates the significance level *p* < 0.05; power of >0.8 represents the probability of detecting if a statistically significant difference exists between the measured variables.
